# Becker’s Nevus in a Male: A Case Report

**DOI:** 10.7759/cureus.45005

**Published:** 2023-09-11

**Authors:** Mridul Bhardwaj, Abhishek Joshi, Swarupa Chakole, Dev B Goel, Atharv Sardesai

**Affiliations:** 1 Medicine, Jawaharlal Nehru Medical College, Datta Meghe Institute of Higher Education and Research, Wardha, IND; 2 Community Medicine, Jawaharlal Nehru Medical College, Datta Meghe Institute of Higher Education and Research, Wardha, IND; 3 Medicine and Surgery, Jawaharlal Nehru Medical College, Datta Meghe Institute of Higher Education and Research, Wardha, IND

**Keywords:** hyper-pigmentation, hamartoma, laser-therapy, becker's melanosis, becker's nevus

## Abstract

Nevus is a group of melanocytes that grow together to form a benign growth on the skin. It is often a black lesion that may protrude from the skin. Becker's nevus is a hyperpigmented lesion that presents congenitally or is acquired. The pigmentation and unusually high hair growth might grow darker with time. Becker's nevus, which can interchangeably be called Becker's melanosis, is a rare disease usually presented by men. The following case is of a 21-year-old male who presented with a hyperpigmented lesion on his right arm. The lesion started at age 16 and increased in size gradually; it involved the flexor surface of the right elbow joint and showed hypertrichosis with irregular margins. On examination, the top dermis contained melanophages, and the basal layer was hyperpigmented. Based on clinical appearance and examination, Becker's nevus was diagnosed.

## Introduction

In 1949, Becker published the first description of Becker's nevus (BN) [1]. BN is also known as Becker melanosis. It is a benign hamartomatous lesion that can develop from birth or be acquired through hypertrichosis or lesions that cause hair loss. It's a rare illness that mostly impacts men [2]. It is frequently pigmented, grows darker over time, and is frequently covered in profuse hair growth. BN generally manifests as a hyperpigmented patch with wavy edges that gradually expands over a few years before stabilizing [3]. Although not always present, hypertrichosis within a lesion is common. BN syndrome (BNS), alternately known as hairy epidermal nevus syndrome, was initially identified by Happle in 1997. BNS is characterized by various cutaneous as well as musculoskeletal abnormalities, generally ipsilateral [4]. The smooth muscle hamartoma is the most frequent histological association of BN [5]. It is also important to note that BN is the only epidermal nevus that does not adhere to Blaschko lines [6]. Certain Actin-Beta (ACTB) gene mutations in BN may enhance Hedgehog signaling in the smooth muscle of the arrector pili, a tissue derived from the embryonic mesoderm, impairing the formation of hair follicles in a noncell autonomous manner [7]. Given that BN is more likely to result in acne and hypertrichosis, both of which are more common in men, the notion of hyperresponsive hormones (i.e., the sensitivity of androgens) is put out in this cutaneous hamartoma [8]. Rarely congenital, it may manifest in childhood, although it typically comes to light for the first time in puberty. It starts light in hue and then gets more noticeable. Although there have not been many reported cases of bilateral origin, it frequently manifests unilaterally [9].

## Case presentation

**Figure 1:**
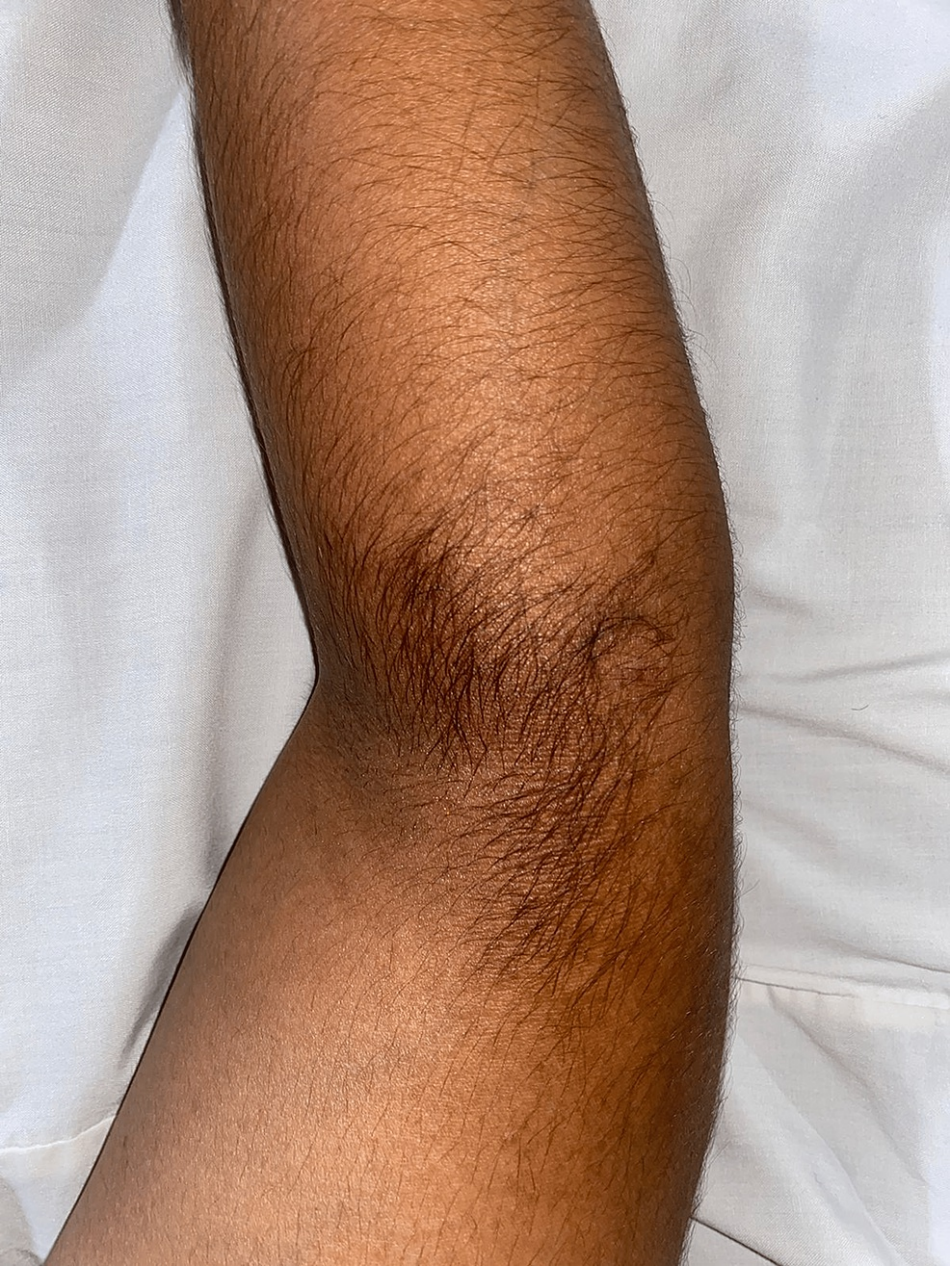
Hyperpigmented lesion on right hand

A 21-year-old healthy medical student from Delhi presented with an asymptomatic hyperpigmented patch on the flexor surface of the elbow of his right arm for the past six years. The patch was small at first and gradually progressed to increase in size before stopping to grow after a year. It is measured 5 x 3cm and is located on the flexor part of the elbow joint with irregular spread but sharp demarcation and even hyperpigmentation throughout (Figure 1). There is a history of hypertrichosis on this patch of skin. There are no provoking or relieving factors involved or identified. There is no history of evolution in size, shape, or color since its initial growth episode. There is no associated history of itching or tenderness. The absence of any systematic symptoms, along with presenting history, was used to rule out any malignant condition. The patient has no history of smoking, alcohol abuse, allergies, or any psychological condition. The patient has no relevant past or family history and does all normal activities for a person of his age.

**Figure 2:**
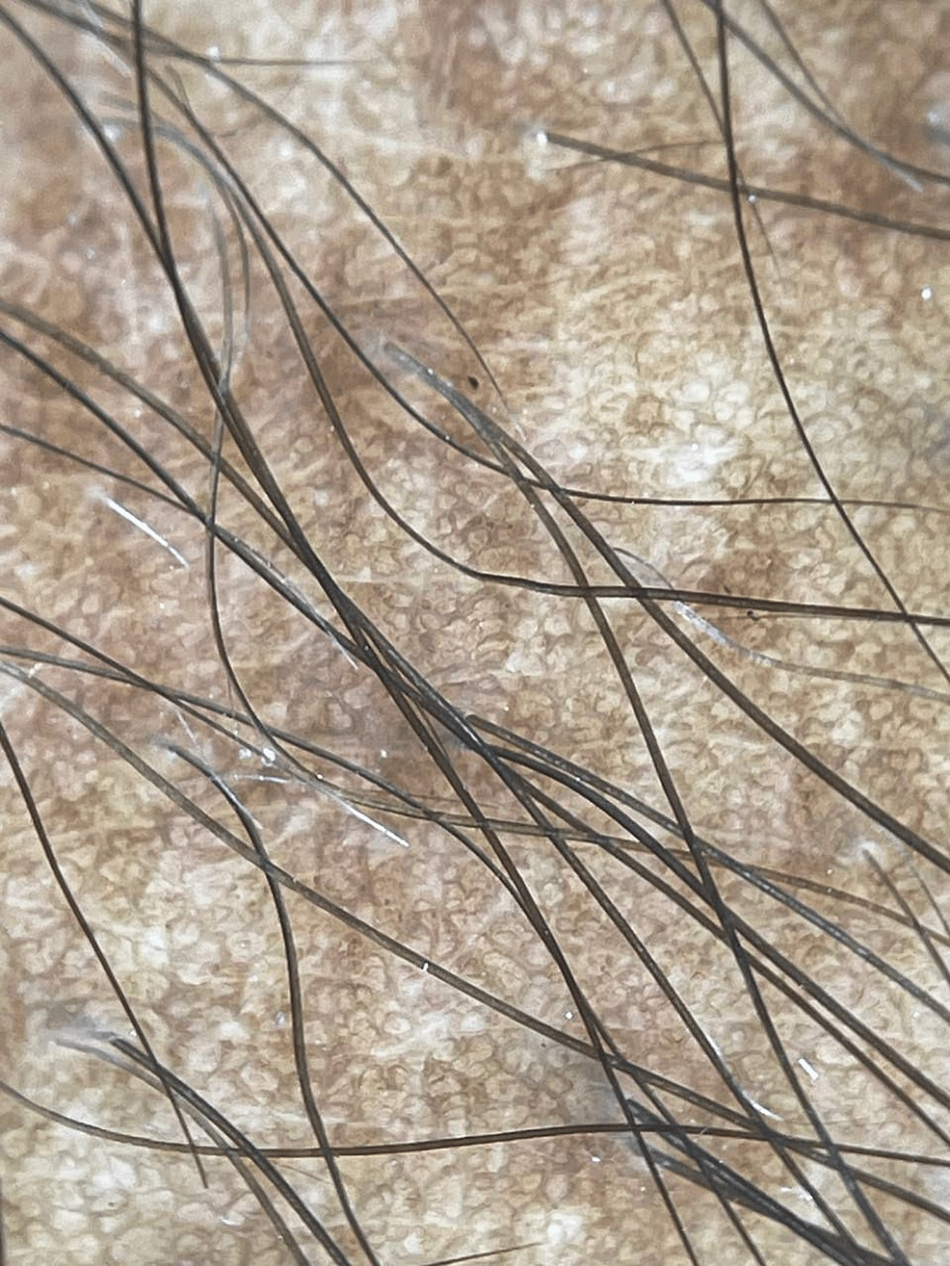
Dermatoscopy showing uniform-sized network of hyperpigmentation with perifollicular hypopigmentation

Dermoscopy examination with a polarizing digital dermatoscope (Figure 2) performed on the lesion demonstrated a well-defined pigment network and uniform thickness of lines. Further uniform size and shape of a network of pigmentation with perifollicular hypopigmentation was observed.

The patient was counseled about the cosmetic hindrance posed by the lesion and was advised Q-switched laser treatment along with a follow-up; however, since the condition is benign and does not pose any threat, the patient decided not to pursue any line of treatment.

## Discussion

There are various clinical manifestations of BN. Although an autosomal dominant inheritance mode with varying penetration in the skin has been proposed, the acquired disease is most frequently the cause. Research comprising over 19,000 French male recruits between the ages of 17 and 26 found a prevalence of 0.5%. Showing a 5:1 male-to-female ratio, BN is more prevalent in men. Increased androgen receptor density is proof that there is a disruption in the activity of androgen receptors in BN [10]. In various nations, the incidence of BN in men is reported to range between 0.25% and 4.2%. For BN, there are 2:1 to 6:1 more men than women [11]. The variances can be attributed to females' readily apparent breast hypoplasia and the absence of hypertrichosis. Further, the disease may manifest as a complex of developmental abnormalities of various organ systems in addition to a classical epidermal nevi, such a manifestation is referred to as BN syndrome. However, the predominant mode of inheritance, which results in a mosaic population of hemizygous or homozygous cells in otherwise heterozygous individuals, provides a better explanation for the sporadic occurrence of BN [12]. To treat BN, various laser treatments have been employed alone or in conjunction. The number of BN treatments varied from 1 to 12, and the laser wavelengths utilized ranged from 504 to 10,600 nm. Although a combination of lasers with different wavelengths seemed more effective, the clinical results were mixed [13]. Pulsed dye (504 nm), Q-switched Nd:YAG (532 nm), intense pulsed light (550 nm), intense pulsed light (615 nm), and other lasers are employed. Long-pulsed alexandrite laser (755 nm), 808 nm and 810 nm Diode lasers, Q-switched ruby laser (694 nm), Erbium-doped fiber laser (1,550 nm), Q-switched Nd:YAG (1,064 nm), Erbium: YAG (2,940 nm), ablative fractional laser (10,600 nm), Erbium: YAG (2,940 nm) and Q-switched Nd:YAG (1,064 nm), Alexandrite laser (755 nm) with long-pulsed Nd:YAG (1,064 nm), long-pulsed Nd:YAG, Q-switched alexandrite laser (755 nm), non-ablative fractional erbium-doped laser (1,550 nm), and topical hydroquinone [14]. Lasers are generally safe. However, they only have a modest impact on BN. Combination therapy appears to have the potential to enhance the result [15].

## Conclusions

Becker melanosis is another name for BN. It is a benign lesion that can appear congenitally or acquired with hypertrichosis or hairlessness lesions. It's a rare condition that primarily affects men. It is frequently pigmented, grows darker over time, and is frequently covered in profuse hair growth. BN is a rare clinical illness with a high level of anxiety that is primarily diagnosed clinically. Since the disease has received less investigation and writing as compared to other dermatological disorders such cases should be recorded regardless of the presentation being typical or atypical. An extensive amount of research must be performed on the disease regarding the atypical presentations and treatment modalities to ensure prompt diagnosis. As such, diagnosis is rare and needs to be considered. Patients must be reassured since the disease process is usually benign and the prognosis good.
